# Systematically linking tranSMART, Galaxy and EGA for reusing human translational research data

**DOI:** 10.12688/f1000research.12168.1

**Published:** 2017-08-16

**Authors:** Chao Zhang, Jochem Bijlard, Christine Staiger, Serena Scollen, David van Enckevort, Youri Hoogstrate, Alexander Senf, Saskia Hiltemann, Susanna Repo, Wibo Pipping, Mariska Bierkens, Stefan Payralbe, Bas Stringer, Jaap Heringa, Andrew Stubbs, Luiz Olavo Bonino Da Silva Santos, Jeroen Belien, Ward Weistra, Rita Azevedo, Kees van Bochove, Gerrit Meijer, Jan-Willem Boiten, Jordi Rambla, Remond Fijneman, J. Dylan Spalding, Sanne Abeln

**Affiliations:** 1 Department of Computer Science, Vrije Universiteit Amsterdam, Amsterdam, 1081 HV, Netherlands; 2 The Hyve, Utrecht, 3511 MJ, Netherlands; 3 SURFsara, Amsterdam, 1098 XG, Netherlands; 4 ELIXIR Hub, Hinxton, CB10 1SD, UK; 5 Department of Genetics, University Medical Center Groningen, University of Groningen, Groningen, 9712 CP, Netherlands; 6 Department of Bioinformatics, Erasmus University Medical Center, Rotterdam, 3015 CE, Netherlands; 7 EMBL-EBI, Hinxton, CB10 1SD, UK; 8 Netherlands Cancer Institute, Amsterdam, 1066 CX, Netherlands; 9 Dutch Techcentre for Life Sciences, Utrecht, 3521 AL, Netherlands; 10 Department of Pathology, VU University Medical Center Amsterdam, Amsterdam, 1081 HV, Netherlands; 11 Lygature, Utrecht, 3521 AL, Netherlands; 12 Centre for Genomic Regulation (CRG), Barcelona, 08003, Spain

**Keywords:** tranSMART, EGA, Galaxy, FAIR, reproducibility, translational research, data management, workflows

## Abstract

The availability of high-throughput molecular profiling techniques has provided more accurate and informative data for regular clinical studies. Nevertheless, complex computational workflows are required to interpret these data. Over the past years, the data volume has been growing explosively, requiring robust human data management to organise and integrate the data efficiently. For this reason, we set up an ELIXIR implementation study, together with the Translational research IT (TraIT) programme, to design a data ecosystem that is able to link raw and interpreted data. In this project, the data from the TraIT Cell Line Use Case (TraIT-CLUC) are used as a test case for this system. Within this ecosystem, we use the European Genome-phenome Archive (EGA) to store raw molecular profiling data; tranSMART to collect interpreted molecular profiling data and clinical data for corresponding samples; and Galaxy to store, run and manage the computational workflows. We can integrate these data by linking their repositories systematically. To showcase our design, we have structured the TraIT-CLUC data, which contain a variety of molecular profiling data types, for storage in both tranSMART and EGA. The metadata provided allows referencing between tranSMART and EGA, fulfilling the cycle of data submission and discovery; we have also designed a data flow from EGA to Galaxy, enabling reanalysis of the raw data in Galaxy. In this way, users can select patient cohorts in tranSMART, trace them back to the raw data and perform (re)analysis in Galaxy. Our conclusion is that the majority of metadata does not necessarily need to be stored (redundantly) in both databases, but that instead FAIR persistent identifiers should be available for well-defined data ontology levels: study, data access committee, physical sample, data sample and raw data file. This approach will pave the way for the stable linkage and reuse of data.

## Introduction

Translational research, or translational medicine, sets out to translate novel biological insights into clinical diagnostic tools, medicine, procedures, policies and education ^1, 2^. Recent developments in high-throughput profiling techniques like next generation sequencing ^3^, followed by third generation sequencing ^4^ and the earlier techniques like tandem mass spectrometry ^5^ and microarrays ^6^, have revolutionised translational research. Raw data generated by these techniques require extensive computation by bioinformatics workflows ^7^, which transform raw data into interpreted data. The impressive number of observables per sample (e.g. genes, transcripts, exon positions, or peptide fragments) indicates that we need more samples to enhance the statistical power in filtering relevant biological events; moreover, it is still expensive to generate new molecular profiling data for research ^8^. Subsequently, there is an increasing need to be able to reuse patient-derived high-throughput molecular profiling data from existing studies. The clinical and pathological information of such samples should also be stored to allow reanalysis. Additionally, all of these data are privacy sensitive, and hence require careful storage and controlled access. Here, we describe how those needs can be implemented into a well-designed data management ecosystem for archiving, linking and reusing data to facilitate the data-driven translational research on a large scale.

**Figure 1.**
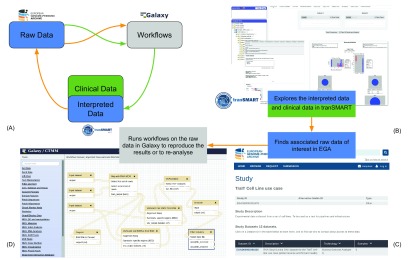
The usage scenario considered in the implementation study. **A**: The process for data generation (green lines) is different from that for data reuse (orange lines). **B**– **D**: Intended scenario of reusing data for translational research: first, the samples of interest can be discovered by exploring the clinical and interpreted data in tranSMART (v16.1); note that it is essential to present enough metadata for effective exploration ( **B**); next, the raw data in EGA can be traced back from the interpreted data in tranSMART ( **C**); finally, workflows can be re-applied to the raw data in Galaxy ( **D**). **A**: The process for data generation (green lines) is different from that for data reuse (orange lines). **B**– **D**: Intended scenario of reusing data for translational research: first, the samples of interest can be discovered by exploring the clinical and interpreted data in tranSMART (v16.1); note that it is essential to present enough metadata for effective exploration ( **B**); next, the raw data in EGA can be traced back from the interpreted data in tranSMART ( **C**); finally, workflows can be re-applied to the raw data in Galaxy ( **D**).

We consider two potential usage scenarios: 1) the process associated with generating the data; and 2) the process associated with reusing previously generated data. Note that the starting point in the two processes are different: in the former, the user starts by storing and computationally processing the raw data from the high-throughput experiments (green lines in Figure 1:A), whereas the latter process naturally starts from exploring, analysing or querying the interpreted data (orange lines in Figure 1:A).

Many previous initiatives have focused on the implementation of infrastructures for processing and storing previously generated data ^9– 11^, but few focus on the scenario of reusing the data. Several consortia currently provide data infrastructures aimed to enable life science research ^12– 15^. Moreover, various initiatives have pushed the idea to make scientific results and data more openly accessible ^16– 19^. In light of this, a joint effort between ELIXIR and TraIT has been established to set up an implementation study with the aim of designing an ecosystem connecting existing data systems to enable effective reuse of the data. ELIXIR ^20^ is an intergovernmental organisation which builds on existing data resources and services within Europe, enhancing European-wide biological research. Translational research IT (TraIT) is established as a large public-private partnership to develop, implement and maintain a long-lasting IT infrastructure for translational research in the Netherlands. In this work, we describe the setup, results and recommendations of the EGA-TraIT ELIXIR implementation study.

Several resources and databases have been dedicated to store, query, explore, process and analyse human data. In this study, we aim to connect the European Genome-phenome Archive (EGA) ^21^, tranSMART ^10, 22, 23^ and Galaxy ^24, 25^. Currently, tranSMART (v16.1) and Galaxy are deployed by TraIT, while the EGA infrastructure is supported by CRG, EBI and ELIXIR. tranSMART is an open source framework and cloud platform for integrating molecular plus clinical data and exploring these; therefore tranSMART is a natural starting point for reusing data by making data findable. Galaxy is an open source bioinformatics workflow management system ^7, 25^, in which workflows can be run intuitively to analyse the biomolecular profiling raw data by users without programming expertise. The European Genome-phenome Archive (EGA) is a longterm data repository for molecular profiling and phenotypic data, where data are stored, managed, referenced and distributed with strict access control. As of June 2017, more than 1160 studies are available at EGA, with over 8000 data access accounts. It thus has become a highly used archive for raw human translational research data, helping to improve data accessibility.

The intended usage scenario of the implementation study is the reproduction and reanalysis of archived data, and can be outlined as follows: a life science researcher is exploring the interpreted and clinical data in tranSMART ( Figure 1B) to find a few specific samples of interest; they then can retrieve the identifiers for these samples in EGA, and thus retrieve the raw data from EGA ( Figure 1C), and (re)apply computational workflows made available through Galaxy ( Figure 1D).

**Figure 2.**
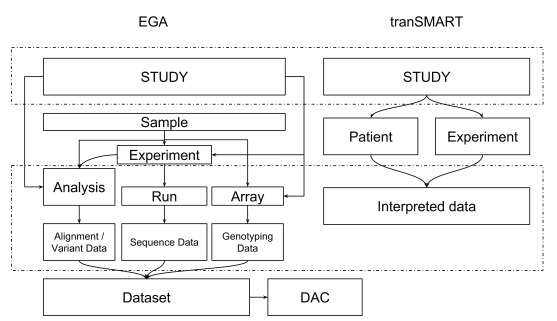
Metadata mapping between EGA and tranSMART. The data model of EGA is dramatically different from that of tranSMART (v16.1) due to the deviating purposes and designs of the systems. Furthermore, in both systems, there is an intrinsic flexibility in defining the data model. EGA uses the SRA (sequence read archive) data model for NGS data with the addition of array data from array and genotyping experiments. EGA also exports all sample objects to BioSamples, ensuring each sample has a BioSample ID. tranSMART focuses on the clinical information and interpreted biomolecular profiling data. The data model has a patient-centered, but flexible structure which also shows some design choices due to the underlying relational database. Terminology is not the same between tranSMART and EGA - partially due to the SRA data model employed at EGA, such that an experiment describes the library and platform used for sequencing experiments only. In tranSMART, a wider range of experiments can be described. DAC is a data access committee. The sample level, which is lacking in tranSMART v16.1, will be supported from v17.1.

Here we report the full outcome of this implementation study; previously, we described the connection between Galaxy and EGA ^26^. In this paper, we show a proof of concept that demonstrates the feasibility of linking data resources for reusing archived data, with the help of the TraIT Cell Line Use Case (TraIT-CLUC) data. Nevertheless, the dramatic differences in data models between data resources, like EGA and tranSMART ( Figure 2), have posed a major challenge for the interoperability of linking data. We finalise this work with a recommendation on how to transform the proof of concept into a mature solution. We show how to bridge the distinct data models of the different data sources by using persistent identifiers (PID), and explain how this befits the FAIR ^16^ use of human data and computational workflows in translational research: findable, accessible, interoperable and reusable.

## Results and discussion

### Data ecosystem design

**Figure 3.**
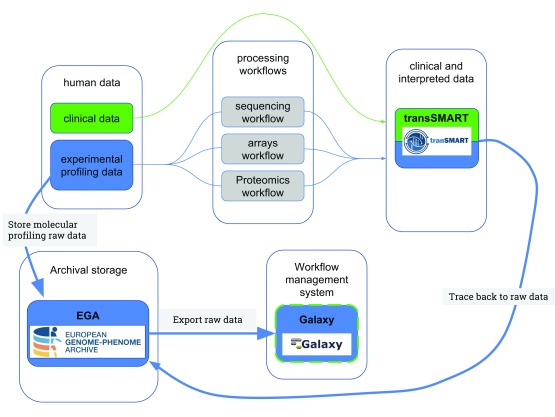
A designed data ecosystem based on TraIT: interpreted data together with clinical data can be explored in tranSMART, subsequently tracked back to the raw data in EGA, and finally, raw data in EGA can be imported to Galaxy, where workflows can be applied to the raw data. The blue arrows in this figure depict the connections implemented as a proof of concept by the current work.

We designed a data ecosystem in this implementation study connecting part of the TraIT infrastructure with EGA, as shown in Figure 3; in this figure, the blue arrows show the links implemented in this study. Note that we emphasise the process for reusing data here, starting from the interpreted data in tranSMART, linking back to the raw data in EGA that can be imported within Galaxy. Galaxy can subsequently be used to rerun the workflows over the raw data or perform novel analyses.

### ELIXIR implementation proof of concept

The TraIT Cell Line Use Case (TraIT-CLUC) raw data, which are non-privacy sensitive, were made public in EGA. Via the EGA help desk, anyone can access them for testing and developing workflows.

**Figure 4.**
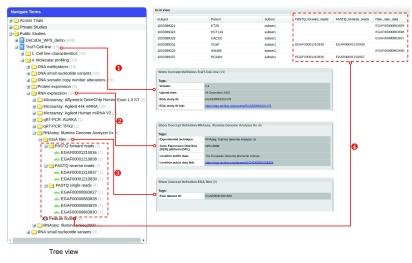
An implementation in tranSMART (v16.1) of metadata mapping between tranSMART and EGA. (1): The study level mapping; if one hovers over the node ‘TraIT-Cell-line’ study node, one can see the EGA study identifier. (2) and (3): Metadata of node “EGA files” and its parent node (e.g.“RNA expression”) in the tree view contains one EGA dataset ID that those EGA file IDs (i.e. the leaf nodes of “EGA files”) belong to (dataset in EGA is similar to series in GEO). (4): After dragging the node “EGA files” in the tree view to ‘Grid View’, raw data files with EGA File IDs are rendered in a few columns in ‘Grid View’, where each row stands for a mapping from the interpreted data to its corresponding raw data files. Each subnode (not leaf node) of node “EGA files” in the tree view corresponds to a column in ‘Grid View’. Therefore, the interpreted data in tranSMART can be traced back to the corresponding raw data archived in EGA, either via the corresponding files or via the entire dataset.

With the TraIT-CLUC data, we showcase an implementation of data model mapping between tranSMART and EGA ( Figure 4), which enables the envisioned data reuse process. Users in tranSMART can: trace back all the interpreted data in one study to all the raw data file IDs by EGA study ID, which is in the metadata of the study in tranSMART - (1) in Figure 4.

1. trace back all the interpreted data under one specific experiment type to the raw data file IDs by the EGA Dataset ID. The EGA Dataset ID can be found in the metadata of node "EGA files" and its parent node (e.g."RNA expression") in the tree view - (2) and (3) in Figure 4.
2. trace back one piece of specific interpreted data under one specific experiment type to the raw data files by EGA file IDs, which are the leaf nodes of the node ‘EGA files’ in the tree view and rendered as columns in ‘Grid View’ - (4) in Figure 4.

Once the users in tranSMART retrieve EGA file IDs, they can directly import the raw data files into a Galaxy instance with the Galaxy tool “EGA download streamer” ^26^. Subsequently, the workflow in Galaxy can be applied to these data for reproduction or new analysis.

### Implemented improvements to EGA

During the upload of the TraIT-CLUC data, there had been extensive communication and feedback between the TraIT and EGA team. This has resulted in an improved data uploading pipeline. EGA has implemented a FUSE layer, which allows all files received from EGA via the downloader to be stored in an encrypted format on the remote filesystem. This also allows processes to natively access these files and decrypt them automatically as they are accessed, removing the need for a separate specific decryption step and hence the storage of unencrypted files on a remote filesystem and the associated security concerns. This implementation is now being extended to allow remote file transfer to remote clouds. 

In order to improve the findability of data stored in EGA, a draft API has been implemented which allows objects to be queried and filtered, with the response in JSON format. The objects to return are specified, followed by the object and ID to filter by. For example, the following query returns the datasets associated with study EGAS00001001476: https://test.ega-archive.org/metadata/v2/datasets?queryBy=study&queryId= EGAS00001001476. It is also possible to retrieve the BioSample and EGA IDs of the samples associated with the study using the following query: https://test.ega-archive.org/metadata/v2/samples? queryBy=study&queryId=EGAS00001001476&limit=0.

The current work has improved the level of FAIRness of the infrastructure in several ways. The findability (F), even though in this case of a controlled access database, has been improved by generating a link back to the raw data. The accessibility (A), in this case with controlled access, has also been improved by allowing data import using EGA identifiers in Galaxy to access the raw data, making it thereby reusable (R). The main challenge in the implementation study is the interoperability (I), i.e., the data model mapping between EGA and tranSMART, which are unsurprisingly different from each other ( Figure 2). Below we outline recommendations to further improve the FAIRness of this ecosystem for privacy sensitive human data.

## Recommendation to implement a proof of concept

In this ELIXIR EGA-TraIT implementation study, we showed a proof of concept for linking EGA, tranSMART and Galaxy, effectively providing an ecosystem for translational high-throughput biomolecular profiling data. However, the current implementation of metadata mapping between tranSMART and EGA will become more cumbersome when one item of interpreted data corresponds to multiple raw data files, which leads to multiple columns in the “grid view” of tranSMART. In this situation, to allow the further development of technical links, user-friendly interfaces, better provenance of computational methods and a more structural solution is required. Below we will outline our recommendations, which will ensure interoperability between different elements of these ecosystems, and thus allow the development of user-friendly work processes.

### Recommendations to move from a proof of concept to a mature solution

The ELIXIR implementation study aimed to show a proof of concept for a functioning ecosystem, in which data could be reused by life science researchers. In order to make a user-friendly, and more mature ecosystem, some further improvements need to be made:

1. The current implementation of the Galaxy EGA download streamer means that all users of one Galaxy instance have to share one user credential to access EGA data. Currently, Galaxy does not support password input type. This means that any password will be inadvertently recorded in the Galaxy history, and thereby compromise the security of EGA credentials; the current implementation is an ad hoc solution to this problem. A generic solution in Galaxy should be offered to securely integrate with the third-party authentication ^27^; this would also enable secure personal access to nonpublic databases besides EGA.
2. From a user perspective, error messages from the Galaxy EGA download streamer should be easily interpretable. Currently, it is difficult to obtain associated metadata on the EGA file identifiers, making it difficult to implement helpful error messages. For example, it may be unclear to the user why there is no access to a certain file, and who should be approached if access is needed. This could be addressed if metadata on EGA identifiers would be exposed in a more generic, machine readable format, preferably in RDF.
3. Likewise, human readable metadata associated with EGA identifiers, such as the file identifier, should be exposed, so that researchers can find their way to the correct datasets, studies and data access committees covering the files of interest. Currently, if a life science researcher finds an EGA file ID in tranSMART, and does not have EGA access yet, it is very difficult to find out to which EGA dataset or study it belongs.
4. For life science researchers, a more direct reference from tranSMART to suitable computational workflows would be highly desirable. In terms of provenance, a reference to the workflow that produced the data would be sufficient; however, for reusing data by the life science researchers, it would be helpful if a direct link to a workflow on a Galaxy instance were available. This issue has for example been addressed in the myFAIR Analysis project.
5. Many bioinformaticians running production workflows for generating interpreted data do not, in fact, use Galaxy. An important reason for this is that Galaxy does not always give enough control over the data usage and job scheduling to allow computationally expensive workflows to be run efficiently on HPC systems. Moreover, a bioinformatician — who wants to make a Galaxy workflow available as provenance over the dataset and increase reusability of the data — needs to make additional efforts to port the workflow to Galaxy. Any steps that make this porting easier, will in the longer term greatly serve the provenance of interpreted data.

### Recommendations to systematically link data resources for human data

Currently, data models used to capture clinical cohorts vary strongly between different data resources ( Figure 2). However, aligning these data models, or mapping them via metadata, would only partially resolve the problem for the following reasons:

1) Translational research is a rapidly changing field; study and cohort structures rapidly evolve to reflect the fast advances in data science and high-throughput molecular profiling techniques.

2) Different elements within any such ecosystem can have multifarious purposes and can aim to serve a different market of users.

3) Metadata is essential for good data stewardship ^16^; nevertheless, the purposes of data resources may indicate which metadata is required; moreover, metadata may need to be corrected or updated over time (see for example the fate of the TCGA barcodes).

4) Making huge amounts of (overlapping) metadata a requirement in each data resource will increase the barrier for data submission to any resource.

**Figure 5.**
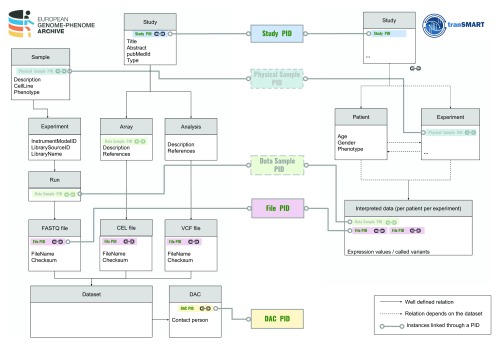
Suggested usage of persistent identifiers to link concepts between the data models of EGA and tranSMART. The data model of EGA differs much from that of tranSMART; for example, a tranSMART experiment has a different conceptual meaning compared to the EGA ‘experiment’, which is one of the four ‘processing’ objects at EGA (experiment, run, analysis, and array). A few well-defined entities with persistent identifiers (PIDs) are essential to achieving the interoperability between the systems. From this implementation study, Study PID, File PID and DAC PID are thought to be essential for systematic mapping for a stable ecosystem allowing to reuse data. Moreover, from a TraIT perspective, stable identifier types that describe the physical sample (Physical Sample PID) and the raw data associated with such a sample (Data Sample PID) are desirable. For the first concept, the BioSample definition could be used, for the second concept, it is clear that there is a need for a well-defined aggregate identifier above the file level that covers all raw output data from a single experiment on a single sample. Ongoing studies aim to generate a well-defined level for these needs, which are also consistent with GA4GH ^29^ metadata model systems.

In this context, we make a different suggestion that ensures interoperability between these systems without the need to align their full relational structures: globally resolvable and unique persistent identifiers (PID) ^28^ should be generated for well-defined entities in all data resources, and should be used to link the data between data resources ( Figure 4). Furthermore, we suggest that following ontology concepts need to be assigned such persistent identifiers: Study, Data Access Committee (DAC), Physical Sample, Data Sample, and Data File ( Figure 5).

We suggest the following requirements should hold for each of these persistent identifiers:

1. A single authority should be responsible for minting the persistent identifier, which also entails a scheme to define what the string looks like, and for standardising minimally required metadata applied for the identifier within the consortium.
2. Any data resource offering these PIDs should make sure the relations between the PID entities are resolvable by querying their database, for those PIDs included in the resource. For example, if EGA contains a File PID, we should be able to ask for the associated DAC PID.

Such persistent identifiers would be very similar to the recently introduced ORCID ID for researchers. Several data resources, as held by publishers, libraries and funding agencies, are including this in their systems, which obviates the need for a homogeneous relational structure or perfectly overlapping metadata. The linkage of one ORCID ID with multiple DOIs makes the publications and academic activities of one researcher easily traceable, creating a fully workable researcher-centered ecosystem with a wide range of data resources and applications.

Note that it is not necessary for all types of PIDs to be governed by a single authority. Currently, EGA has two types of PIDs listed at identifiers.org: the EGA study and EGA Dataset. All EGA samples also have a BioSamples PID, which links to the publicly accessible attributes of the sample. To fully adhere to the above criteria, EGA would need to ensure that the controlled-access attributes are available via an EGA PID, along with EGA PIDs for Experiment, Analysis, Run, and Array. The additional PID types required may also be given out by other authorities; distributed governance of PID types would not diminish their usefulness.

With our recommendations, this implementation study specific data ecosystem will further progress towards FAIR guiding principles. If the associated metadata of these PIDs are made available as linked data, the findability (F) could easily be ensured by metadata exposing systems such as bioschemas ^30^ or wikidata ^31^; in this way, users could easily access the metadata and PIDs in Wikipedia via search engines. A file PID or Data Sample PID should be associated with at least one DAC PID, ensuring that high-throughput biomolecular profiling data can be authorised and accessed (A). The implementation of PIDs in linking metadata specifically achieves the interoperability (I) between different systems. Raw data in EGA can be reused in Galaxy for further analysis in our data ecosystem and the rich metadata will help users evaluate the reusability (R) of the data. The latter will be enhanced if our recommendation can push the regulation of the community standard in human data management. Thus, we suggest that by determining a few well-defined entities in a rigorous way, we can link existing initiatives, built with different purposes in mind, without the need for aligning their full data structures.

### Ongoing implementation of a FAIRpoint system for EGA

EGA has traditionally only allowed a limited set of data to be available publicly because of its controlled-access database. These would be the study, DAC, and dataset objects. This study has shown that for EGA to become fully FAIR, EGA needs to allow all other objects with PIDs to be publicly queryable. EGA can ensure security by restricting the attributes of the PIDs that are visible publicly, but allow the PID itself to be public. For example, as each file in EGA has a PID, this PID could be public, while the filename could be under controlled access, allowing the full structure and links between objects at EGA to be accessible. EGA is developing a new API that will allow the relationships between all objects to be determined (linked data) while ensuring controlled access data is not public. Example queries would be:

- ’List all files from sample A’
- ’List all samples used in file B’
- ’List all files of type C in study D’
- ’List all samples in dataset E’
- ’Return the experiments that were performed on sample F by study G’

Additionally, filters can be applied to restrict results by attributes associated with an object, such as ’Return all BAM files from male samples in study H’. EGA should also extend the extant relevant digital objects listed at identifiers.org ^30^ for each of which EGA is responsible for generating a PID, ensuring that each of these objects will have a unique uniform resource identifier (URI).

## Conclusions

Our implementation study advances the role of EGA from a data archive towards a data port, where data can more readily be reused; additionally, our implementation study has made it possible to link tranSMART, Galaxy and EGA into a full data reuse ecosystem. Interoperability is the centrepiece among all the challenges in linking data and our recommendation offers one solution to it. In addition, this implementation study allowed us to make several recommendations for future projects to improve FAIRness of the designed ecosystem.

## Methods

### Data model mapping

We map the data model of tranSMART (v16.1) and that of EGA. In Figure 2, “study” in both databases are mapped; “interpreted data” is mapped to “analysis” or “run” in EGA which corresponds to one or multiple EGA file IDs (see the section "Data and software availability").

### TraIT-CLUC data

TraIT-CLUC data are used in this implementation study for test purposes because they do not have privacy issues. TraIT-CLUC data include results obtained from various high-throughput molecular profiling techniques, such as microarrays, next generation sequencing and tandem mass spectrometry. Raw data were restructured to be uploaded into EGA; the interpreted data were rendered as the tranSMART-ready format to be uploaded into tranSMART (see *Data and software availability*).

### Data uploading and publishing

***Data upload into EGA.*** Raw TraIT-CLUC data including FASTQ and BAM files were uploaded into EGA together with their metadata.

Data files were transferred to EGA archival via FTP after being encrypted locally. Metadata were filled into XML files and uploaded into EGA via its API. The raw TraIT-CLUC data have been structurally published in EGA.

***Data upload into tranSMART.*** The interpreted TraIT-CLUC tranSMART-ready data were uploaded into tranSMART using transmart-batch ^32^.

### EGA data into Galaxy

A Galaxy tool called ega_download_streamer ^26^ was used, which wraps EGA download client into Galaxy. We set up an EGA account with access to TraIT-CLUC data into Galaxy. By providing an EGA file identifier, this tool enables the automatic download of data from EGA into Galaxy.

## Data and software availability

### TraIT-CLUC data

The raw TraIT-CLUC data structurally published in EGA can be accessed via EGA Study ID EGAS00001001476. These data are public and therefore anyone can request the access to the datasets under EGA Study ID EGAS00001001476 via EGA help desk (DAC ID: EGAC00001000514). The access to the tranSMART-ready TraIT-CLUC interpreted data can be found at https://trng-b2share.eudat.eu/records/21bdc3128e1541da83dc48c51cd39a5f. How to load the tranSMART-ready data into tranSMART can be found at http://cluc.trait-platform.org.

### tranSMART

tranSMART (v16.1) is used in this implementation study. Information about a demo server of tranSMART showcasing the data model mapping of this work can be found at http://cluc.trait-platform.org.

### Galaxy

A Galaxy instance can be deployed either from the source code or from a Docker image. More information can be found at https://galaxyproject.org/. Galaxy tool “EGA download streamer” can be installed from the main Galaxy tool shed under the name “ega_download_streamer” within the Galaxy instance. The source code can be found at http://dx.doi.org/10.5281/zenodo.167330 ^33^.
