# Supplementary material for: Systematically linking tranSMART, Galaxy and EGA for reusing human translational research data
Source: F1000Res. 2017 Aug 16;6:ELIXIR-1488. [Version 1] doi: 10.12688/f1000research.12168.1 (PMC5657030; doi:10.12688/f1000research.12168.1)
Supplement: Peer review history

# Peer review: Referee response for version 1

**DOI:** 10.5256/f1000research.13170.r27264

**Published:** 2017-10-24

**Authors:** Gilbert S. Omenn

**Affiliations:** 1 Department of Internal Medicine, School of Public Health, University of Michigan, Ann Arbor, MI, USA

This is a quite unusual paper.  It presents a demo with TraIT of a cell line use case (CLUC) to combine functions, samples, and datasets  from tranSMART (v16.1) and EGA into workflows in Galaxy.  I was pleased to see proteomics identified as a key data type in Figure 3.

 Figure 4 shows the metadata mapping and the assessment of the FAIR principles.

 The Discussion of the ELIXIR implementation lays out improvements needed.  Sometimes we might require a manuscript to report the implemented improvements, with results, but in this complex situation, including recommendations for managing public access versus controlled access across different independent resources, I think indexing at this stage is worthy.  The model of ORCID IDs for researchers and their publication DOI's is a useful analogy.

I have read this submission. I believe that I have an appropriate level of expertise to confirm that it is of an acceptable scientific standard.

# Peer review: Referee response for version 1

**DOI:** 10.5256/f1000research.13170.r25072

**Published:** 2017-09-06

**Authors:** Hervé Ménager

**Affiliations:** 1 Centre de Bioinformatique, Biostatistique et Biologie Intégrative (C3BI, USR 3756 Institut Pasteur et CNRS), Paris, France

This paper describes the work implemented in the context of an ELIXIR implementation study, which aims at building a proof of concept for an infrastructure that links reference omics data (from the EGA) with a workflow environment (Galaxy) and a data integration platform hosting interpreted data (transMART).

 The authors make a clear case of showing the interest of their approach, which is to facilitate the discovery and reusability (overall, the FAIRness) of clinical data. A prototype “ecosystem” has been built to evaluate this approach. As the authors mention, this paper builds, among other things, on the work presented in “Integration of EGA secure data access into Galaxy” ^1^, which had also introduced the project. The results of this work are quite encouraging, as the implementation study demonstrates that despite technical issues such as the difference of the data model for different components (EGA and transMART), their integration remains possible.

 The last “Recommendations” section is helpful in understanding the limitations of the current work. Of particular importance in my opinion is recommendation 5 to move to a “mature solution”, which explains the difference of implementation between the initial analysis and the re-analysis workflows by the restriction of Galaxy usage to smaller scales than the “production workflows” used initially. This raises the question of workflow portability between and Galaxy and other workflow management systems. I personally think that CWL ^2^ (an initiative I am currently part of) could be used as a standard language to define workflows which can be run both in high-throughput production environments, and in graphical workbench systems like Galaxy. From a more general perspective, most of the recommendations corresponding to potential modifications in the “partner systems” (EGA, Galaxy, transMART), it would be interesting to know if they have been communicated to the corresponding communities, and to be able to track the evolution of these requests.

 A last minor point is that I would modify figure 3 to transform the “Export raw” between EGA and Galaxy into an “Import raw data”, as the data transfer is controlled from Galaxy rather than from EGA.

I have read this submission. I believe that I have an appropriate level of expertise to confirm that it is of an acceptable scientific standard.

## References

[1] HoogstrateYZhangCSenfABijlardJHiltemannSvan EnckevortDRepoSHeringaJJensterGJ A FijnemanRBoitenJWA MeijerGStubbsARamblaJSpaldingDAbelnS: Integration of EGA secure data access into Galaxy. *F1000Res*.2016;5: 10.12688/f1000research.10221.1 10.12688/f1000research.10221.1 28232859PMC5302147

[2] O'ConnorBDYuenDChungVDuncanAGLiuXKPatriciaJPatenBSteinLFerrettiV: The Dockstore: enabling modular, community-focused sharing of Docker-based genomics tools and workflows. *F1000Res*.2017;6: 10.12688/f1000research.10137.1 52 10.12688/f1000research.10137.1 28344774PMC5333608
